# Exploratory Cost-Effectiveness Analysis of Response-Guided Neoadjuvant Chemotherapy for Hormone Positive Breast Cancer Patients

**DOI:** 10.1371/journal.pone.0154386

**Published:** 2016-04-28

**Authors:** Anna Miquel-Cases, Valesca P. Retèl, Bianca Lederer, Gunter von Minckwitz, Lotte M. G. Steuten, Wim H. van Harten

**Affiliations:** 1 Department of Psychosocial Research and Epidemiology, Netherlands Cancer Institute, Amsterdam, The Netherlands; 2 German Breast Group, Neu-Isenburg, Germany; 3 University of Twente, Department of Health Technology and Services Research, Enschede, The Netherlands; 4 Hutchinson Institute for Cancer Outcomes Research, Fred Hutchinson Cancer Research Center, Seattle, United States of America; University of Torino, ITALY

## Abstract

**Purpose:**

Guiding response to neoadjuvant chemotherapy (guided-NACT) allows for an adaptative treatment approach likely to improve breast cancer survival. In this study, our primary aim is to explore the expected cost-effectiveness of guided-NACT using as a case study the first randomized controlled trial that demonstrated effectiveness (GeparTrio trial).

**Materials and Methods:**

As effectiveness was shown in hormone-receptor positive (HR+) early breast cancers (EBC), our decision model compared the health-economic outcomes of treating a cohort of such women with guided-NACT to conventional-NACT using clinical input data from the GeparTrio trial. The expected cost-effectiveness and the uncertainty around this estimate were estimated via probabilistic cost-effectiveness analysis (CEA), from a Dutch societal perspective over a 5-year time-horizon.

**Results:**

Our exploratory CEA predicted that guided-NACT as proposed by the GeparTrio, costs additional €110, but results in 0.014 QALYs gained per patient. This scenario of guided-NACT was considered cost-effective at any willingness to pay per additional QALY. At the prevailing Dutch willingness to pay threshold (€80.000/QALY) cost-effectiveness was expected with 78% certainty.

**Conclusion:**

This exploratory CEA indicated that guided-NACT (as proposed by the GeparTrio trial) is likely cost-effective in treating HR+ EBC women. While prospective validation of the GeparTrio findings is advisable from a clinical perspective, early CEAs can be used to prioritize further research from a broader health economic perspective, by identifying which parameters contribute most to current decision uncertainty. Furthermore, their use can be extended to explore the expected cost-effectiveness of alternative guided-NACT scenarios that combine the use of promising imaging techniques together with personalized treatments.

## Introduction

Neoadjuvant (preoperative) chemotherapy (NACT) is an option in patients with breast cancer. Equally effective as adjuvant chemotherapy [1,2], this approach allows direct and early observation of treatment response [3]. Based on this response, patient’s further systematic treatment can be tailored, i.e. responders continue with the same initial treatment, and non-responders can be switched to a presumably non-cross resistant regimen. This adaptive treatment approach is likely to improve breast cancer survival.

**Fig 1:**
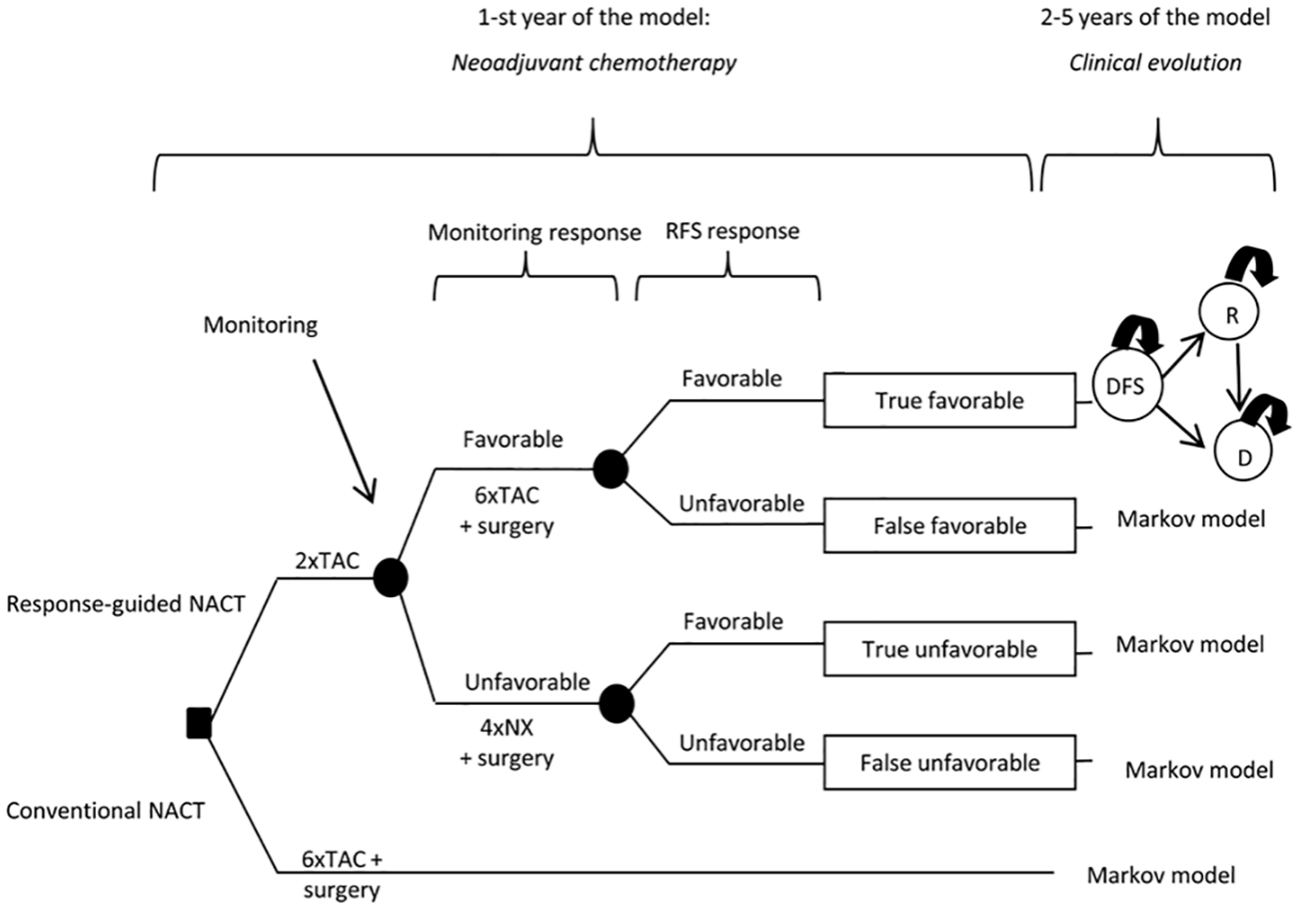
Decision tree and Markov model. Decision nodes (black squares) are points at which the patient or health provider makes a choice. Chance nodes (black circles) are points at which more than one event is possible but is not decided by neither the patient or health provider. During the 1^st^ model cycle, patients receive the intervention; response-guided neoadjuvant chemotherapy (NACT), starting with 2xTAC followed by 4xNX (unfavorable at monitoring) or by 6xTAC (favorable at monitoring), or conventional-NACT, with equal treatment of 6xTAC to all patients, followed by surgery. In the following 4-year cycles, the Markov model simulates the clinical evolution of the patients. *TAC* docetaxel, doxorubicin, and cyclophosphamide, *NX* vinorelbine and capecitabine

The GeparTrio trial [4] presents the first long-term survival results (overall survival; OS and disease free survival; DFS) of guided-NACT in breast cancer. In this trial, 2012 early breast cancer (EBC) women were initially treated with two cycles of docetaxel, doxorubicin, and cyclophosphamide (TAC) followed by response assessment by palpation and ultrasound. Thereafter, patients classified as early responders were randomly assigned to four or six additional TAC cycles, and patients classified as non-responders to four cycles of TAC or four cycles of vinorelbine and capecitabine (NX) before surgery (Fig 1). For the survival analysis the two investigational response-guided arms (8xTAC and 2xTAC/4xNX) were grouped and compared with the conventional therapy arms (6xTAC). No significant differences in OS were observed, however a longer DFS after guided-NACT was seen in the subgroup of hormone-receptor positive (HR+) patients (hazard ratio 5-years DFS = 0.56).

The interpretation of these results is that intensifying the same chemotherapy to respondents, or switching to NX in non-respondents, only works in HR+ patients. While the lack of effectiveness seen in HR-/ human epidermal growth factor receptor 2 positive (HER2+) patients could be justified by the lack of Trastuzumab administration in this trial, in the case of HR-/HER2- patients, this could be consequence of treatment ineffectiveness. There is a large body of evidence suggesting that in this subgroup there may be other treatments beyond chemotherapy [5]. For instance DNA-damaging agents such as platinums, potentiators of DNA damage such as the investigational agent iniparib, and inhibitors of poly-ADP ribose polymerase such as olaparib [6]. Furthermore, capecitabine administered after surgery has been shown to improve outcomes in this group [7].

The results of this study need to be interpreted with caution for several reason: 1) they rely on a secondary exploratory subgroup analysis; 2) they are the first to provide such an indication for guided-NACT and need validation, especially in the context of current therapeutic decision-making (as Trastuzumab was not used); 3) there is no clear understanding of the underlying reason for its single benefit to HR+ patients only (whether that is direct consequence of the cytotoxic effect from the regimes used, or whether that is caused from an indirect endocrine effect causing chemotherapy induced amenorrhea [8,9]; and 4) the evaluation of estrogen-receptor (ER), progesterone-receptor (PgR) and HER2 status was not reassessed centrally, which may also limit the interpretation of the trial results. Our judgment is that this hypothesis needs to be prospectively tested before guided-NACT as investigated in this trial is ready for routine clinical practice in HR+ breast cancer.

If this scenario of guided-NACT proves effective, cost-effectiveness will play a central role in adoption and reimbursement decision-making. Hence, a timely explorative CEA to estimate its expected cost-effectiveness is warranted. This study aims at determining the expected cost-effectiveness of guided-NACT as proposed by the GeparTrio trial using input clinical data from the trial.

## Materials and Methods

### Treatment strategies compared

Two NACT interventions were compared: *Guided-NACT* (as presented in the GeparTrio trial): 2-cycles of docetaxel 75 mg/m^2^, doxorubicin 50 mg/m^2^, and cyclophosphamide 500 mg/m^2^, on day 1 every 3 weeks (2xTAC), followed by monitoring with ultrasound (US) and palpation. Treatment with 6xTAC if patients were favorable respondents at monitoring and four courses of vinorelbine 25 mg/m^2^ on day 1 and 8 plus capecitabine 1.000 mg/m^2^ orally twice a day on day 1 through 14, every 3 weeks (4xNX) if patients were unfavorable respondents at monitoring. Favorable and unfavorable patients were classified following published criteria [10]. In short, favorable response was defined as a “≥50% reduction in the product of the two largest perpendicular diameters of the primary tumor” assessed at the end of the second cycle and before surgery. *Conventional-NACT*: Treatment with 6xTAC without monitoring. Within the same year, all patients underwent surgery (classified as either mastectomy only, or breast-conserving-surgery (BCS) with radiotherapy).

### Model overview

A Markov model (Microsoft Excel 2010, Microsoft Corporation, Redmond, WA) estimated the health-economic consequences of treating 50-years old [10] HR+ EBC women with guided-NACT vs. conventional-NACT. The model with three health-states: disease free (DFS), relapse (R, including local, regional, and distant) and death (D, including breast cancer and non-breast cancer), simulated the clinical evolution of these patients over a time-horizon of 5-years (Fig 1).

Patients entered the model in the DFS health-state, after completing NACT and surgery, classified as true-favorable, true-unfavorable, false-favorable and false-unfavorable respondents of NACT at monitoring (definitions in Table 1). The “gold standard” for NACT response was the 5-years relapse free survival (RFS), as it provides a reasonable threshold to capture all relapses related to NACT response [11].

**Table 1:** Definitions of true-positive, false-positive, true-negative and false-negative used in our study.

Group of patients	Definition
True favourable	Patient that is classified as **favourable** at monitoring, continues receiving 6xTAC, and after 5 years of follow up is classified as **favourable** due to absence of relapse event
False favourable	Patient that is classified as **favourable** at monitoring, continues receiving 6xTAC, and after 5 years of follow up is classified as **unfavourable** due to presence of relapse event
True unfavourable	Patient that is **unfavourable** at monitoring, switches to 4xNX, and after 5 years of follow up is classified as **favourable** due to absence of relapse event (*the underlying assumption is that the patient was not responding to 2xTAC but did to 4xNX*, *thereby demonstrating that monitoring classified the patient properly)*
False unfavourable	Patient that is **unfavourable** at monitoring, switches to 4xNX, and after 5 years of follow up is classified as **unfavourable** due to presence of relapse event (*the underlying assumption is that the patient was responding to 2xTAC and did not to 4xNX*, *thereby demonstrating that monitoring classified the patient wrongly)*

From this DFS health-state, patients could either 1) move to the R health-state, i.e., ‘relapse’; 2) move to the D health-state, i.e., ‘non-breast cancer death’, 3) stay in the DFS health-state, i.e., ‘no event and administration of adjuvant hormonal treatment, assumed to be an aromatase inhibitor (AI)’. During the 1^st^ year of the DFS health state, patients could incur NACT-related toxicities, including heart failure, (febrile) neutropenia, asthenia and alopecia [10]. From the R health-state, patients could either 1) move to the D health-state, i.e., ‘breast cancer related death’; or 2) stay in the R health-state, i.e., ‘cured relapse’. We assumed that patients could only develop one relapse.

In each annual model cycle, patients moved/stayed in one of the mutually exclusive health-states, as explained above, according to transition probabilities (tps). During each year, patients cumulated life-years (LY), quality-adjusted life-years (QALYs), and costs. The costs and health-related quality-of-life (HRQoL) associated to the health-states are presented in Table 2.

**Table 2:** Costs and quality-of-life associated to the Markov model health-states.

Health state	Year cycle	Costs	HRQoL
DFS	1^st^ without NACT related toxicities	NACT and surgery	NACT
DFS	1^st^ with NACT related toxicities	Toxicity/es treatment	Disutility from toxicity
DFS	2^nd^/5^th^	AI	AI
R event	1^st^	Relapse treatment	Relapse
R cured	2^nd^/5^th^	DFS year 2^nd^/5^th^	DFS year 2^nd^/5^th^
D breast cancer	1^st^/5^th^	Palliative treatment	none
D other causes	1^st^/5^th^	none	none

*HRQoL* health related quality of life, *DFS* disease free survival, *R* relapse, *D* death, *NACT* neoadjuvant chemotherapy, *AI* aromatase inhibitors

### Clinical data

The clinical data used to derive tp in our CEA is a subset of previously published data[10]; the group of HR+ patients. Our definition of HR+ was somewhat different from that of the original trial, as we selected positivity of the ER+ only, thus excluding the group of PR+/ ER- patients. This was reasoned by their small proportion among all cases, 92/1295 patients (7%), and by their absence of influence in ER+ prognosis[12,13]. The total number of HR+ patients included in our analysis was of 1203.

From these patients, Kaplan-Meier (KM) curves (IBM SPSS Statistics for Windows, Version 22.0. Armonk, NY: IBM Corp.) of RFS (interval from finishing the NACT intervention to occurrence of first relapse) and breast cancer specific survival (BCSS; interval from relapse to occurrence of breast cancer death) were derived for the group of conventional-NACT patients on one hand (n = 602), and for the combined group of false-favorable and false-unfavorable patients (of the guided-NACT arm) on the other hand (n = 67). No KMs nor tps were calculated for the true-favorable and true-unfavorable (with 100% response on the switch treatment) patients (n = 233), whom by definition do not relapse and thereby do not die from breast cancer. The group of false- favorable/unfavorable and true- favorable/unfavorable were derived by using the 5-year DFS threshold to the total patients receiving response-guided NACT (n = 601). The formula *S*(*t*) = exp^{−*kt*} where *k* is the hazard rate and *t* is time was used to derive the tps of relapse and breast cancer death from the aforementioned KM curves. Patients who suffered from toxicities were assumed to benefit equally from NACT and the same tps were applied. Non-breast cancer deaths were accounted by using age-specific death rates from the Central Bureau of Statistics of the Netherlands [14].

Furthermore, data on the “responsiveness” after 2xTAC, medically significant NACT-related toxicities [15], and type of surgeries was provided. This was included in the model as proportions.

### Quality of life

Utilities (preferences weights) related to model health-states, chemotherapy, AI and heart failure were derived from literature [16–18] based on EuroQoL-5D measures [19]. Utility scores for febrile neutropenia, asthenia and alopecia were derived by subtracting toxicity related dis-utilities in breast cancer [20] to the baseline chemotherapy utility. The same method was used to derive the utility score for neutropenia, but using non-small-cell-lung-cancer literature as a proxy [21] owing to absence of more specific data in the breast cancer literature. Utility scores for both surgery types were assumed equal [22–24]. No literature on the effect of monitoring on HRQoL was found, thus it was assumed unaltered.

### Costs

Costs (€2013) included direct medial and non-medical costs (i.e., traveling costs), and costs of productivity losses (friction cost method [25]). Drug resource use (calculated for patients of 73 Kg and body-surface area of 1.8 m^2;^ anthropometric measurements derived from a population of Swedish women [26]), estimates on direct-non medical costs and costs of productivity losses were derived from the GeparTrio protocol and their unit costs from Dutch sources on costs and prices [27–29] or literature [30,31]. Costs of treating toxicities [32–35], of surgery [36], of radiotherapy [36] and of the model health-states [37] were also derived from literature. Costs of monitoring included one breast examination by palpation (counted as one medical visit) and a sonography [38]. We used exchange currencies [39] when needed, and the consumer price index to account for inflation [40].

Values for tps, HRQoL data and costs are presented in S1 Table.

### Base-case cost-effectiveness analysis

Effects were expressed in LYs and QALYs, costs as mean cost per patient, and cost-effectiveness as the incremental cost-effectiveness ratio (ICER; difference in expected costs/difference in expected QALYs for the guided-NACT vs. conventional-NACT strategy). The ICER was compared to the prevailing Dutch threshold for cost-effectiveness of severe disease (€80.000/QALY) [41]. To facilitate the adoption decision, the ICER was arranged into the net monetary benefit (NMB). If the expected NMB is >0, guided-NACT is cost-effective and a positive adoption recommendation follows [42].

### Probabilistic sensitivity analysis

Uncertainty around the ICER estimate was calculated via probabilistic sensitivity analysis (PSA) with 10.000 second order Monte-Carlo simulations of the model. For the PSA, each model parameter was entered in the model along with a distribution by following the recommendations by Briggs et al [43]. A beta distribution was assigned to binomial data such as toxicities and transition probabilities, a dirichlet distribution to the proportions of true/false favourable/unfavourable patients, and a gamma distribution to rightly skewed data such as costs (S1 Table). We discounted future costs and health effects at a 4% and 1.5% yearly rate respectively, according to the Dutch guidelines on health-economics evaluations [29]. Results were reported in cost-effectiveness acceptability curves (CEAC), which reflect the probability of each alternative to be cost-effective at a range of threshold values for cost-effectiveness.

### One-way sensitivity analysis

We performed a one-way sensitivity analysis (SA) to all model parameters by varying them within one standard deviation of error or, a 25% of their base case value if this information was missing, and observed its effect on the NMB.

## Results

### Base-case cost-effectiveness analysis

We predicted with our model that guided-NACT prevents 1.210 relapses and 102 breast cancer deaths in 10.000 treated patients over a period of 5-year. This translated into 0.011 LYs and 0.014 QALYs gained. Furthermore, we observed that while switching response to 4xNX only added €6.379, continuing with 6xTAC added €22.797. Differences came from a combination of high drug costs in the TAC regimen (highest costs per cycle: T = €1198 and pegfilgrastim = €1161), vs the NX regimen (highest costs per cycle: N = €227 and X = €180), and a higher frequency of costly adverse events. Favorable respondents (8xTAC) were the most costly patients, followed by conventionally treated patients (6xTAC) and unfavorable respondents (2xTAC/4xNX). Overall, guided-NACT was more expensive than conventional-NACT due to having 65% of patients assigned to 8xTAC. However, as this was more effective than conventional-NACT, the resulting discounted ICER was cost-effective (€7.737/QALY, under a €80.000/QALY, corresponding with a NMB of €1.025).

### Probabilistic sensitivity analysis

**Fig 2:**
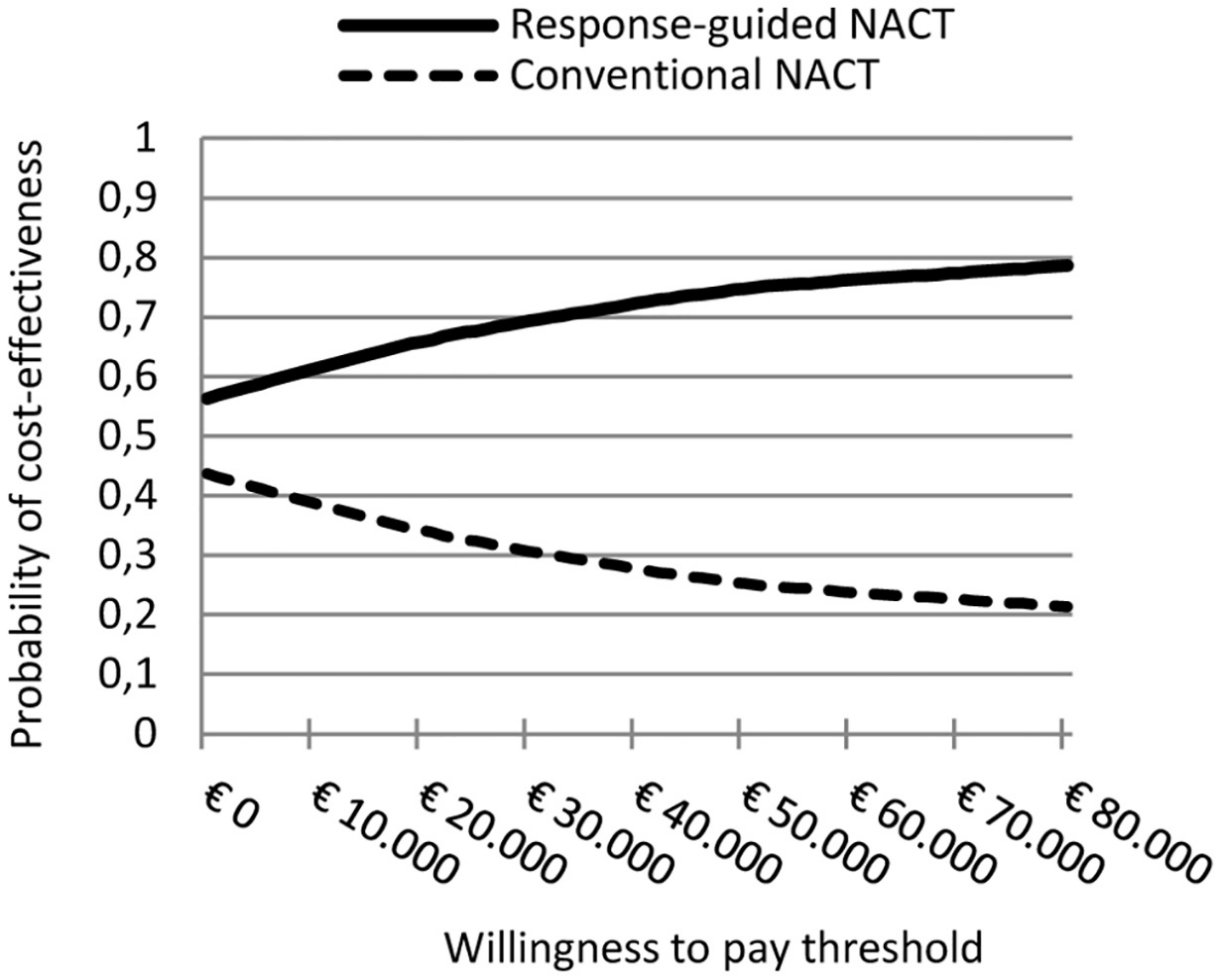
Cost-effectiveness acceptability curves. They show the probability of response-guided neoadjuvant chemotherapy (NACT) and conventional-NACT of being cost-effective at different levels of willingness-to-pay threshold (WTP). At WTP thresholds below €80.000/QALY, response-guided NACT had a higher probability of being cost-effective, ranging from 60% at €10.000/QALY to 78% at the Dutch WTP threshold for severe diseases of €80.000/QALY

The CEAC showing the cost-effectiveness of guided-NACT at different willingness to pay thresholds is presented in Fig 2. This shows that guided-NACT is expected cost-effective at any willingness to pay per additional QALY. At the Dutch willingness to pay threshold of €80.000/QALY, guided-NACT was expected cost-effective with 78% certainty.

Results for the base-case CEA and the PSA are presented in Table 3.

**Table 3:** Results of the base-case cost-effectiveness analysis and the probabilistic sensitivity analysis.

Base-case CEA									PSA
Strategy	Costs(€)	LY	QALY	ΔLY	ΔQALY	Δcosts	ICER (€/QALY)	NMB(€)	Prob. (%)
Guided-NACT	81.904	4,717	3,324	0,011	0,014	€110	7.737	1.025	78
Conventional- NACT	81.795	4,706	3,310	-	-	-	-	-	22

*CEA* Cost-effectiveness analysis, *LY* Life years, *QALY* Quality adjusted life years, *ICER* Incremental cost-effectiveness ratio, *NMB* Net monetary benefit, *PSA* Probabilistic sensitivity analysis

### Sensitivity analysis

**Fig 3:**
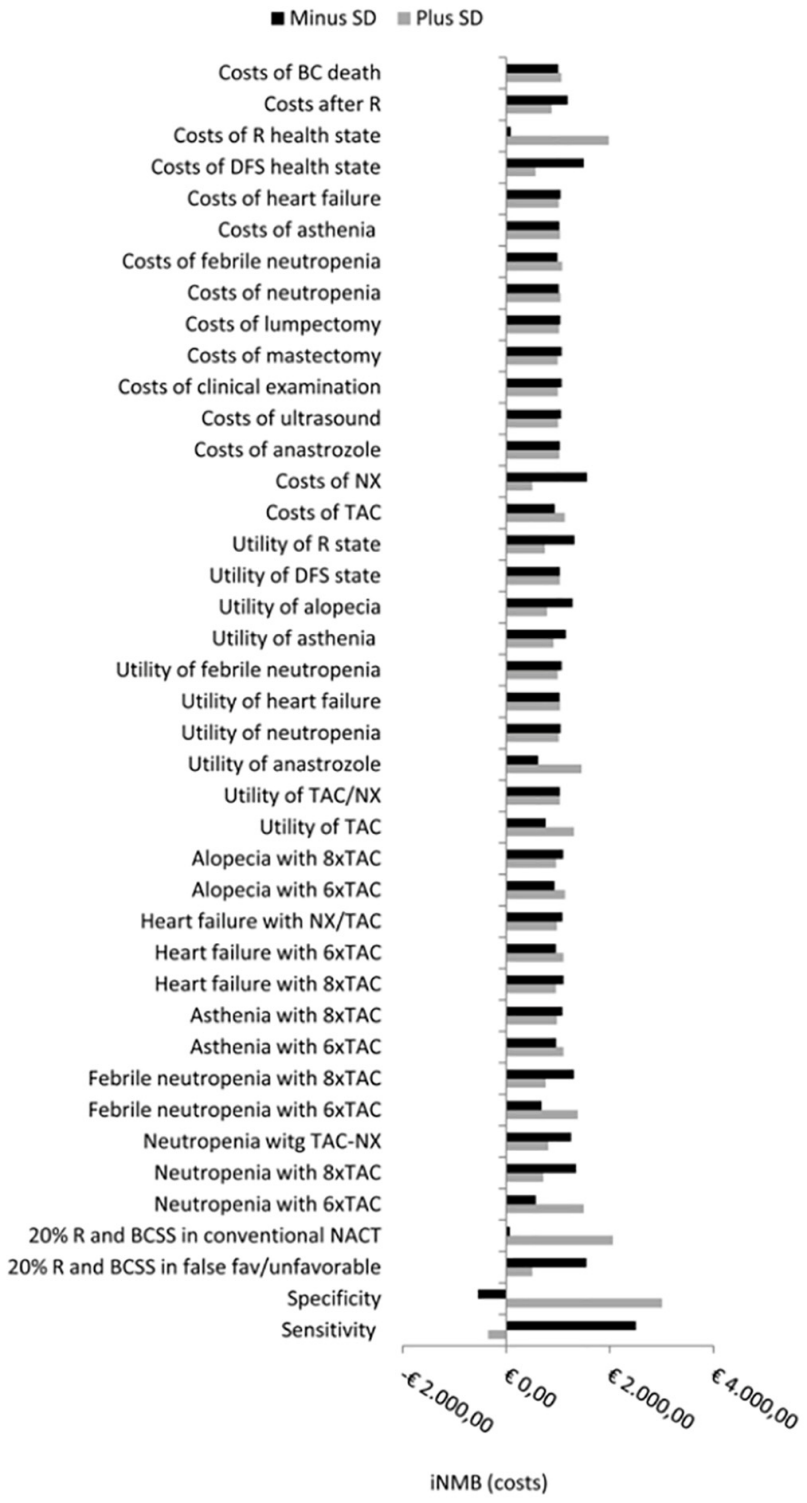
One-way sensitivity analysis to all model parameters. We explored how varying model parameter values could affect the net monetary benefit (NMB). If this became negative, it means that response guided neoadjuvant chemotherapy became cost-ineffective. The NMB remained cost-effective at all parameters values tested, except at specificity of 55% and sensitivity of 100%, were the NMB became negative. Furthermore, an increase in the proportion of relapses and deaths in the conventional-NACT strategy, an increase in the costs of the R health-state and a decrease in the costs of NX markedly increased cost-effectiveness.

In one-way SA, the NMB remained cost-effective at all parameters values tested, except at low specificity values (55%) and high sensitivity values (100%), were the NMB became negative. Furthermore, an increase in the proportion of relapses and deaths in the conventional-NACT strategy, an increase in the costs of the R health-state and a decrease in the costs of NX markedly increased cost-effectiveness (Fig 3).

## Discussion

Response-guided NACT is a new approach to breast cancer treatment that could improve survival. The first RCT to demonstrate this was the GeparTrio trial. It showed that guiding versus not guiding NACT improved the 5-year survival of HR+ EBC with a HR of 0.56. Although this trial was limited by several reasons (that we listed in the introduction) and requires prospective validation before it can be considered for use in routine clinical practice, it provides the first example of guided-NACT in breast cancer.

The results of our study suggest that guided-NACT as proposed by the GeparTrio trial is expected to be cost-effective (compared to conventional-NACT) at any willingness to pay threshold. This means that its additional €1.100.000 are expected to be outweighed by the prevention of 1.210 relapses and 102 breast cancer deaths in 10.000 treated patients over a period of 5-years. At a specific Dutch threshold for cost-effectiveness of €80.000/QALY, the probability that guided-NACT was cost-effective was of 78%. We are not aware of other cost-effectiveness studies on guided-NACT. Our results can therefore not yet be compared to other estimates.

The observed higher incremental gain in terms of QALYs than LYs (0.014 and 0.011) was explained by a higher proportion of relapsed patients (with lower HRQoL) in the conventional-NACT compared to the guided-NACT strategy (2.372 vs. 1.162). These differences were evidently driven by the HR of the GeparTrio trial that suggested that guiding NACT reduced cancer-related events to half of those observed with conventional NACT. In terms of costs, we observed that the additional €1.100.000 of guided-NACT were consequence of having 65% of patients assigned to 8xTAC, the most costly regimen of the model. Costs were higher in the 8xTAC regimen, followed by the 6xTAC regimen and 2xTAC/4xNX regimen. This order was an aftereffect of the differential costs between Docetaxel and Capectiabine (Docetaxel is ~100 times higher than that of Capectiabine of NX regimen) combined with the frequency of costly adverse events in the TAC regimens. As 35% of patients in the guided-NACT strategy received the low costs and presumably effective 2xTAC/4xNX regimen, it seems reasonable to assume that this contributed to guided-NACT cost-effectiveness.

Our one-way SA identified monitoring performance as the main driver of cost-effectiveness, as this was the only parameter that lead to cost-ineffectiveness. The NMB became negative at low specificity values and at high sensitivity values. This was mainly consequence of an increase of patients that received the costly treatment TACx8 i.e., true-favorable patients at high sensitivities and false-favorable patients at low specificities. Optimal performance requires a trade-off between sensitivity and specificity. Given false-favorable patients are the patients that neither benefit from TACx2 nor TACx6, while receiving the most costly treatment, in this intervention specificity should be prioritized. Recent literature has shown that MRI and PET/CT have shown promising results in this respect i.e., sensitivities and specificities of 68% and 91, and 84%-71% respectively [44,45].

Other parameters influenced the magnitude of cost-effectiveness. For example, the lower values of conventional NACT effectiveness and the lower costs of NX. These are interesting observations to explore in further cost-effectiveness studies. These can show what happens to cost-effectiveness of guided-NACT if different imaging modalities and targeted alternatives [46] are used, and are compared to different regimens. These type of biomarker-driven guided-NACT scenarios [47–49] are expected to entail higher costs, yet their effectiveness is also expected superior [50–54]. While awaiting for evidence to emerge on them [55–57], we advocate embarking on early stage CEAs [58], as the one we have presented here. These CEAs can be used to explore via SA the effects of interactions between model parameters in cost-effectiveness. In turn, these can help identifying those scenarios that are expected to be most cost-effective for each patient subgroup, thereby guiding researchers’ translational efforts on imaging and drug development.

The results of this study are specific to the guided-NACT scenario as described by the GeparTrio trial. As this is the first study that shows the effectiveness of this NACT approach using this specific chemotherapeutic regimens, it is fundamental that this evidence is further validated before any final conclusions on the cost-effectiveness of this guided-NACT scenario can be reached.

Our decision model has limitations of data availability and assumptions. Data availability was a shortcoming for two reasons: 1) when patients had to be split according to monitoring and survival outcomes, that resulted in too small sample sizes to derive reliable KM curves, and it required merging patient groups. Nonetheless, as survival modifiers like age or hormone-receptor status were homogenous in the population, we do not expect relevant survival differences if the analysis had been done separately; 2) when estimating HRQoL, as this was absent in the GeparTrio trial and had to be collected from various, sometimes suboptimal, literature sources. Our model assumptions included the inclusion of radiotherapy costs only after BCS, following recommendations by the National Institutes of Health Consensus panel on early breast cancer [59]; and the restrictive inclusion of NACT-related toxicities to frequencies ≥10%, as less frequent events were assumed to not significantly alter costs and HRQoL. Last, a limitation of the response-guided approach itself was the impossibility to distinguish in the false-favorable group, the patients truly falsely classified at monitoring from the patients irresponsive to 4xNX or NACT in general. Nonetheless, as this is a direct consequence of the use of guided-NACT, it was included as such in the model.

## Conclusion

Guided-NACT (as proposed by the GeparTrio trial) is expected cost-effective in treating HR+ EBC women. While prospective validation of the GeparTrio findings is advisable from a clinical perspective, early CEAs can be used to prioritize further research from a broader health economic perspective, by identifying which parameters contribute most to current decision uncertainty. Furthermore, their use can be extended to explore the expected cost-effectiveness of alternative guided-NACT scenarios that combine the use of promising imaging techniques together with personalized treatments.

## Supporting Information

S1 Table Baseline model data on proportions, survival and costs. (DOCX)

## Abbreviations

AI: Aromatase inhibitor
BCS: Breast conserving surgery
BCSS: Breast cancer specific survival
CEA: Cost-effectiveness analysis
CEAC: Cost-effectiveness acceptability curves
D: Death
DFS: Disease free survival
EBC: Early breast cancer
ER: Estrogen receptor
HER2: Human epidermal growth factor receptor 2
HR: Hormone receptor
HRQoL: Health related quality of life
ICER: Incremental cost-effectiveness ratio
KM: Kaplan Meier
LY: Life years
NACT: Neoadjuvant chemotherapy
NMB: Net monetary benefit
NX: Vinorelbine and capecitabine
OS: Overall survival
PR: Progesterone receptor
PSA: Probabilistic sensitivity analysis
QALY: Quality adjusted life year
R: Relapse
RCT: Randomized controlled trial
RFS: Relapse free survival
SA: Sensitivity analysis
TAC: Docetaxel, doxorubicin, and cyclophosphamide
WTP: Willingness to pay
