# Supplementary material for: Exploratory Cost-Effectiveness Analysis of Response-Guided Neoadjuvant Chemotherapy for Hormone Positive Breast Cancer Patients
Source: PLoS One. 2016 Apr 28;11(4):e0154386. doi: 10.1371/journal.pone.0154386 (PMC4849576; doi:10.1371/journal.pone.0154386)
Supplement: S1 Table — (DOCX) [file pone.0154386.s001.docx]

**S1 Table 1**

Baseline model data on proportions, survival and costs

| Parameter | | | | | mean | | SD | Distribution | Source |
| --- | --- | --- | --- | --- | --- | --- | --- | --- | --- |
| **Proportions** | | | | | | | | | |
|  | Responsiveness | | | | | | | | |
|  |  | | True favorable | | | 0,510 | 0,031 | Dirichlet | ^4^ |
|  |  |  | True unfavorable | | | 0,137 | 0,059 | Dirichlet | ^4^ |
|  |  |  | False favorable | | | 0,267 | 0,043 | Dirichlet | ^4^ |
|  |  |  | False unfavorable | | | 0,087 | 0,026 | Dirichlet | ^4^ |
|  | Surgery | | | | | | | | |
|  |  | | True favorable undergoing lumpectomy | | | 0,655 | 0,040 | beta | ^4^ |
|  |  |  | True unfavorable undergoing lumpectomy | | | 0,679 | 0,052 | beta | ^4^ |
|  |  |  | False favorable undergoing lumpectomy | | | 0,568 | 0,072 | beta | ^4^ |
|  |  |  | False unfavorable undergoing lumpectomy | | | 0,360 | 0,094 | beta | ^4^ |
|  |  |  | Conventional-NACT undergoing lumpectomy | | | 0,636 | 0,020 | beta | ^4^ |
|  | Toxicities (>10% incidence ^a^) | | | | | | | | |
|  |  | | Neutropenia | TACx6 | | 0,421 | 0,019 | beta | ^52^ |
|  |  |  |  | TACx8 | | 0,483 | 0,019 | beta | ^52^ |
|  |  |  |  | TAC/NX | | 0,235 | 0,024 | beta | ^52^ |
|  |  |  | Febrile neutropenia | TACx6 | | 0,074 | 0,010 | beta | ^52^ |
|  |  |  |  | TACx8 | | 0,103 | 0,012 | beta | ^52^ |
|  |  |  | Asthenia | TACx6 | | 0,118 | 0,012 | beta | ^52^ |
|  |  |  |  | TACx8 | | 0,154 | 0,014 | beta | ^52^ |
|  |  |  | Heart failure | TACx6 | | 0,009 | 0,004 | beta | ^52^ |
|  |  |  |  | TACx8 | | 0,006 | 0,002 | beta | ^52^ |
|  |  |  |  | TAC/NX | | 0,007 | 0,005 | beta | ^52^ |
|  |  |  | Alopecia | TACx6 | | 0,104 | 0,012 | beta | ^52^ |
|  |  |  |  | TACx8 | | 0,115 | 0,012 | beta | ^52^ |
| **Transition probabilities** | | | | | | | | | |
| Relapse | | | | | | | | | |
|  | | False favorable/unfavorable | | Tp1 | | 0,069 | 0,031 | beta | ^4^ |
|  |  |  |  | Tp2 | | 0,092 | 0,035 | beta | ^4^ |
|  |  |  |  | Tp3 | | 0,156 | 0,044 | beta | ^4^ |
|  |  |  |  | Tp4 | | 0,243 | 0,052 | beta | ^4^ |
|  |  |  |  | Tp5 | | 0,243 | 0,052 | beta | ^4^ |
|  |  | True favorable/unfavorable | | Tp1, tp2, tp3, tp4 and tp5 | | 0,000 | NA | fixed |  |
|  |  | Conventional NACT | | Tp1 | | 0,038 | 0,008 | beta | ^4^ |
|  |  |  |  | Tp2 | | 0,072 | 0,010 | beta | ^4^ |
|  |  |  |  | Tp3 | | 0,070 | 0,010 | beta | ^4^ |
|  |  |  |  | Tp4 | | 0,059 | 0,010 | beta | ^4^ |
|  |  |  |  | Tp5 | | 0,059 | 0,010 | beta | ^4^ |
| Breast cancer death | | | | | | | | | |
|  | | False favorable/unfavorable | | Tp1 | | 0,000 | NA | fixed | assumption |
|  |  |  |  | Tp2 | | 0,001^b^ | 0,004 | beta | ^4^ |
|  |  |  |  | Tp3 | | 0,049 | 0,026 | beta | ^4^ |
|  |  |  |  | Tp4 | | 0,055 | 0,028 | beta | ^4^ |
|  |  |  |  | Tp5 | | 0,090 | 0,035 | beta | ^4^ |
|  |  | Conventional NACT | | Tp1 | | 0,000 | NA | fixed | assumption |
|  |  |  |  | Tp2 | | 0,016 | 0,011 | beta | ^4^ |
|  |  |  |  | Tp3 | | 0,008 | 0,008 | beta | ^4^ |
|  |  |  |  | Tp4 | | 0,030 | 0,015 | beta | ^4^ |
|  |  |  |  | Tp5 | | 0,083 | 0,024 | beta | ^4^ |
| **Utilities** | | | | | | | | | |
|  | TAC | | | | | 0,620 | 0,039 | beta | ^22^ |
|  | NX | | | | | 0,620 | 0,039 | beta | ^22^ |
|  | Anastrozole | | | | | 0,774 | 0,049 | beta | ^22^ |
|  | Neutropenia | | | | | 0,530 | 0,015 | beta | ^25^ |
|  | Heart failure II & IV | | | | | 0,594 | NA | beta | ^21^ |
|  |  | | Heart failure III | | | 0,590 | 0,020 | beta | ^21^ |
|  |  |  | Heart failure IV | | | 0,505 | 0,049 | beta | ^21^ |
|  | Febrile neutropenia | | | | | 0,470 | 0,085 | beta | ^24^ |
|  | Asthenia | | | | | 0,505 | 0,099 | beta | ^24^ |
|  | Alopecia | | | | | 0.506 | 0.099 | beta | ^24^ |
|  | Relapse | | | | | 0,732 | 0,031 | beta | ^22^ |
|  | Disease free survival | | | | | 0,935 | 0,020 | beta | ^22^ |

| **Costs** | | | | | | | | | | | | | |
| --- | --- | --- | --- | --- | --- | --- | --- | --- | --- | --- | --- | --- | --- |
| Parameter | |  | | | | | Unit costs | Unit measure | Mean resource use | Mean cost | SD ^c^ | Distribution | Source |
| Chemotherapy/Hormone therapy | | | | | | | | | | | | | |
|  | TAC | Dir. Med (total) | | | | |  |  |  | €3.044 | €761 | Gamma | ^53,54^ |
|  |  |  | | Docetaxel | | | €959 | 108 mg | 1.25 | €1.198 | €300 | Gamma | ^53^ |
|  |  |  |  | Doxorubicin | | | €205 | 90 mg | 1 | €205 | €51 | Gamma | ^53^ |
|  |  |  |  | Cyclophosphamide | | | €45 | 1080 mg | 0.83 | €38 | €9 | Gamma | ^32^ |
|  |  |  |  | Day care | | | €279 | Day | 1 | €279 | €70 | Gamma | ^53^ |
|  |  |  |  | Oncologist’s visit | | | €109 | Visit | 1 | €109 | €27 | Gamma | ^53^ |
|  |  |  |  | Dexamethasone IV | | | €3 | 5 mg | 4 | €14 | €3 | Gamma | ^53^ |
|  |  |  |  | Dexamethasone OA | | | €1 | 10 mg | 5 | €3 | €1 | Gamma | ^53^ |
|  |  |  |  | Ciprofloxacin | | | €0.1 | 500 mg | 9 | €1 | €0.3 | Gamma | ^54^ |
|  |  |  |  | Ondasentron ^d^ | | | €6 | 8 mg | 6 | €36 | €9 | Gamma | ^53^ |
|  |  |  |  | Pegfilgrastim | | | €1161 | 1 mg | 1 | €1161 | €290 | Gamma | ^54^ |
|  |  | Dir. Non-Med | | | | | €3 | Day | 1 | €3 | €1 | Gamma | ^32^ |
|  |  | Prod. Loss | | | | | €251 | Day | 3 | €753 | €188 | Gamma | ^32^ |
|  |  | **Total** | | | | |  |  |  | **€3.800** | **€950** | **Gamma** | ^32,53,54^ |
|  | NX | Dir. Med (total) | | | | |  |  |  | €836 | €209 | Gamma | ^53^ |
|  |  |  | | Vinorelbine | | | €91 | 36 mg | 2.50 | €227 | €57 | Gamma | ^53^ |
|  |  |  |  | Capecitabine | | | €16 | 4.500 mg | 11.20 | €180 | €45 | Gamma | ^53^ |
|  |  |  |  | Day care | | | €279 | Day | 1 | €279 | €70 | Gamma | ^32^ |
|  |  |  |  | Oncologist’s visit | | | €109 | Visit | 1 | €109 | €27 | Gamma | ^53^ |
|  |  |  |  | Dexamethasone IV | | | €3 | 5 mg | 1.60 | €5 | €1 | Gamma | ^53^ |
|  |  |  |  | Ondansetron | | | €6 | 8 mg | 6 | €36 | €9 | Gamma | ^53^ |
|  |  | Dir. Non-Med | | | | | €3 | Day | 2 | €6 | €1 | Gamma | ^32^ |
|  |  | Prod. Loss | | | | | €251 | Day | 3 | €753 | €188 | Gamma | ^32^ |
|  |  | **Total** | | | | |  |  |  | **€1.595** | **€399** | **Gamma** | ^32,53^ |
|  | Anastrozole (1 year) | | Dir. Med (total) | | | |  |  |  | €193 | €48 | Gamma | ^53,55^ |
|  |  |  |  | | Anastrozole | | €0.05 | 20 mg/day | 365 | €18.38 | €4.59 | Gamma | ^53^ |
|  |  |  |  |  | Dexa scan | | €174 | Scan | 1 | €174 | €44 | Gamma | ^55^ |
|  |  |  | Dir. Non-Med | | | | €3 | Day | 1 | €3 | €1 | Gamma | ^32^ |
|  |  |  | Prod. Loss | | | | €251 | Day | 1 | €251 | €63 | Gamma | ^32^ |
|  |  |  | **Total** | | | |  |  |  | **€447** | **€112** | **Gamma** | ^32,53,55^ |
| Monitoring | | | | | | | | | | | | | |
|  | Ultrasound | | Dir. Med (total) | | | |  |  |  | €215 | €54 | Gamma | ^40^ |
|  |  |  |  | | Hospital costs | | €163 | Scan | 1 | €163 | €41 | Gamma | ^40^ |
|  |  |  |  |  | Specialists fees | | €52 | Scan | 1 | €52 | €13 | Gamma | ^40^ |
|  |  |  | Dir. Non-Med | | | | €3 | Day | 1 | €3 | €1 | Gamma | ^32^ |
|  |  |  | Prod. Loss | | | | €251 | Day | 0.125 | €31 | €8 | Gamma | ^32^ |
|  |  |  | **Total** | | | |  |  |  | **€ 250** | **€62** | **Gamma** | ^32,40^ |
|  | Clinical examination | | Dir. Med | | | | €109 | Visit | 1 | €109 | € 27 | Gamma | ^53^ |
|  |  |  | Dir. Non-Med | | | | €3 | Day | 1 | €3 | €0.8 | Gamma | ^32^ |
|  |  |  | Prod. Loss | | | | €251 | Day | 0.125 | €31 | €8 | Gamma | ^32^ |
|  |  |  | **Total** | | | |  |  |  | **€143** | **€36** | **Gamma** | ^32,53^ |
| Surgery | | | | | | | | | | | | | |
|  | Mastectomy | | Dir. Med | | | | €21.451 | Surgery | 1 | €21.451 | €1.188 | Gamma | ^57^ |
|  |  |  | Dir. Non-Med | | | | €3 | Day | 1 | €3 | €1 | Gamma | ^32^ |
|  |  |  | Prod. Loss | | | | €251 | Day | 15 | €3.763 | €941 | Gamma | ^32^ |
|  |  |  | **Total** | | | |  |  |  | **€25.217** | **€6.304** | Gamma | ^32,57^ |
|  | Lumpectomy and radiotherapy | | Dir. Med | | | | €21.508 | Surgery and radiotherapy ^e^ | 1 | €21.508 | €403 | Gamma | ^57^ |
|  |  |  | Dir. Non-Med | | | | €3 | Day | 26 | €78 | €19 | Gamma | ^32^ |
|  |  |  | Prod. Loss | | | | €251 | Day | 40 | €10.036 | €2.509 | Gamma | ^32^ |
|  |  |  | **Total** | | | |  |  |  | **€ 31.622** | **€ 7.905** | **Gamma** | ^32,57^ |
| Chemotherapy related toxicities | | | | | | | | | | | | | |
|  | Neutropenia | | Dir. Med | | | | €22.672 | Episode | 1 | €22.672 | €5.668 | Gamma | ^58^ |
|  |  |  | Dir. Non-Med | | | | €3 | Day | 1 | €3 | €1 | Gamma | ^32^ |
|  |  |  | Prod. Loss | | | | €251 | Day | 9 | €2.258 | €656 | Gamma | ^32^ |
|  |  |  | **Total** | | | |  |  |  | **€24.932** | **€6.233** | **Gamma** | ^32,58^ |
|  | Febrile neutropenia | | Dir. Med | | | | €28.690 | Episode | 1 | €28.699 | €7.175 | Gamma | ^58^ |
|  |  |  | Dir. Non-Med | | | | €3 | Day | 1 | €3 | €1 | Gamma | ^32^ |
|  |  |  | Prod. Loss | | | | €251 | Day | 10.7 | €2.685 | €1.229 | Gamma | ^32^ |
|  |  |  | **Total** | | | |  |  |  | **€31.387** | **€7.847** | **Gamma** | ^32,58^ |
|  | Asthenia | | Dir. Med | | | | €1.083 | Episode | 1 | €1.083 | €271 | Gamma | ^59^ |
|  |  |  | Dir. Non-Med | | | | €3 | Day | 2 | €6 | €1 | Gamma | ^32^ |
|  |  |  | Prod. Loss | | | | €251 | Day | No reported. | €976 | €244 | Gamma | ^59^ |
|  |  |  | **Total** | | | |  |  |  | **€2.065** | **€516** | **Gamma** | ^32,59^ |
|  | Heart failure ^f^ | | Dir. Med | | | | €31.528 | Episode | 1 | €31.528 | €7.882 | Gamma | ^60^ |
|  |  |  | Dir. Non-Med | | | | €3 | Day | 1 | €3 | €1 | Gamma | ^32^ |
|  |  |  | Prod. Loss | | | | €251 | Day | 6 | €1.505 | €376 | Gamma | ^60^ |
|  |  |  | **Total** | | | |  |  |  | **€33.036** | **€8.259** | **Gamma** | ^32,60^ |
| Health states | | | | | | | | | | | | | |
|  | Disease free state ^g^ | | Dir. Med (total) | | | |  |  |  | €2.872 | €583 | Gamma | ^61^ |
|  |  |  |  | | In & out –patient^9^ | | €2.793 | Episode | 1 | €2.793 | €563 | Gamma | ^61^ |
|  |  |  |  |  | Drugs | | € 79 | Episode | 1 | € 79 | €20 | Gamma | ^61^ |
|  |  |  | Prod. Loss. ^h^ | | | | €251 | Day | 9.4 | €2.352 | €588 | Gamma | ^61^ |
|  |  |  | **Total** | | | |  |  |  | **€5.225** | **€1.306** | **Gamma** | ^61^ |
|  | Relapse state ^g^ | | *Local relapse* | | | | | | | | | | |
|  |  |  |  | | Dir. Med (total) | | **-** | **-** | **-** | €14.833 | €3.708 | Gamma | ^61^ |
|  |  |  |  |  |  | In & out -patient | €12.497 | Episode | 1 | €12.497 | € 1.692 | Gamma | ^61^ |
|  |  |  |  |  |  | Drugs | €2.336 | Episode | 1 | €2.336 | € 584 | Gamma | ^61^ |
|  |  |  |  |  | Prod. Loss | | €251 | Day | 32.5 | €8.154 | €2.038 | Gamma | ^61^ |
|  |  |  | Total local relapse | | | |  |  |  | €22.987 | €5.747 | Gamma | ^61^ |
|  |  |  | *Distant metastasis* | | | | | | | | | | |
|  |  |  |  | | Dir. Med (total) | | **-** | **-** | **-** | €17.417 | €4.354 | Gamma | ^61^ |
|  |  |  |  |  |  | In & out -patient | €11.645 | Episode | 1 | €11.645 | €1.346 | Gamma | ^61^ |
|  |  |  |  |  |  | Drugs | €5.772 | Episode | 1 | €5.772 | €1.443 | Gamma | ^61^ |
|  |  |  |  |  | Prod. Loss | | €251 | Day | 23.5 | €5.896 | €1.475 | Gamma | ^61^ |
|  |  |  |  |  | Total distant metastasis | |  |  |  | €23.313 | €5.828 | Gamma | ^61^ |
|  |  |  | **Total** | | | |  |  |  | **€23.150** | **€5.787** | **Gamma** | ^61^ |
|  | Breast cancer death state ^g^ | | Dir. Med | | | | €8.296 | Episode | 1 | €8.296 | €2.074 | Gamma | ^61^ |
|  |  |  | Prod. Loss. ^i^ | | | | €251 | Day | 23.5 | €5.896 | €1.474 | Gamma | ^61^ |
|  |  |  | **Total** | | | | **-** | **-** | **-** | €14.192.25 | €3.548 | Gamma | ^61^ |

*SD* standard deviation*, Dir. Med* direct medical costs, *IV* intravenous, *OA* oral administration, *TAC* docetaxel, doxorubicin, and cyclophosphamide, *NX* vinorelbine and capecitabine, *tp* transition probabilities, *NA* not applicable, *Dir. Non-Med* direct non-medical costs, *Prod. Loss* costs of productivity losses

^a^ Febrile neutropenia in 6x TAC was also included, although incidence was of 7,4%

^b^ This tp was zero, but to assign a distribution to it we assigned a baseline value

^c^ If it was missing from the data source we used 25% SD as recommended in Briggs et al ^13^

^d^ We selected this 5-HT3-Antagonist, but others could also be used

^e^ Standard radiotherapy, which consists of 25 cycles of 5 grey

^f^ Calculated as an average of grade III and IV toxicities

^g^ Source did not report travelling expenses thus were not added

^h^ Costs of productivity losses were calculated by using resource use of Lidgren et al ^61^ but with the friction method, as recommended by the Dutch guidelines

^i^ Loss of productivity was assumed to be the same as in the metastatic state
